# Novel *ADA2* Compound Heterozygous Mutations Resulting in Deficiency of Adenosine Deaminase 2 in a Pair of Siblings

**DOI:** 10.1007/s10875-021-00981-0

**Published:** 2021-01-30

**Authors:** Li Guo, Jun Wang, Xinghui Yang, Rongjun Zheng, Natalie Deuitch, Panfeng Tao, Qing Zhou

**Affiliations:** 1 Department of Rheumatology Immunology & Allergy, Children’s Hospital, National Clinical Research Center for Child Health, Zhejiang University School of Medicine, 3333 Binsheng Road, Hangzhou, 310052 China; 2 Life Sciences Institute, Zhejiang University, 866 Yuhangtang Road, Hangzhou, 310058 China; 3 Department of Radiology, Children’s Hospital, National Clinical Research Center for Child Health, Zhejiang University School of Medicine, 3333 Binsheng Road, Hangzhou, 310052 China; 4 Metabolic, Cardiovascular and Inflammatory Disease Genomics Branch, National Human Genome Research Institute (NHGRI), Bethesda, MD USA

To the Editor,

The deficiency of adenosine deaminase 2 (DADA2) is an autoinflammatory disease caused by biallelic loss-of-function mutations in the *ADA2* gene. Approximately 200 cases have been reported worldwide [1] since it was first described in 2014 [2, 3]. While the exact prevalence of DADA2 is unknown, it could be as high as 4:100,000 [4]. DADA2 is a clinically heterogeneous disease, and phenotypes include a spectrum of vasculopathy/vasculitis, immune dysregulation, and/or hematologic abnormalities [4–6]. The most common pathogenic variants of ADA2 are p.G47R and p.R169Q [6, 7]. However, the pathogenesis of DADA2 is still not clear. Tumor necrosis factor (TNF) inhibitors have been proven to be highly effective in treating vasculitic forms of DADA2 [4, 8]; however, they seem to be less effective when it comes to cases manifesting as immunodeficiency or bone marrow failure [4]. In some cases, hematopoietic stem cell transplantation (HSCT) can be beneficial for individuals with bone marrow failure, severe immunodeficiency, or for patients who do not respond to TNF inhibition [4, 6].

## Case Report

**Fig. 1:**
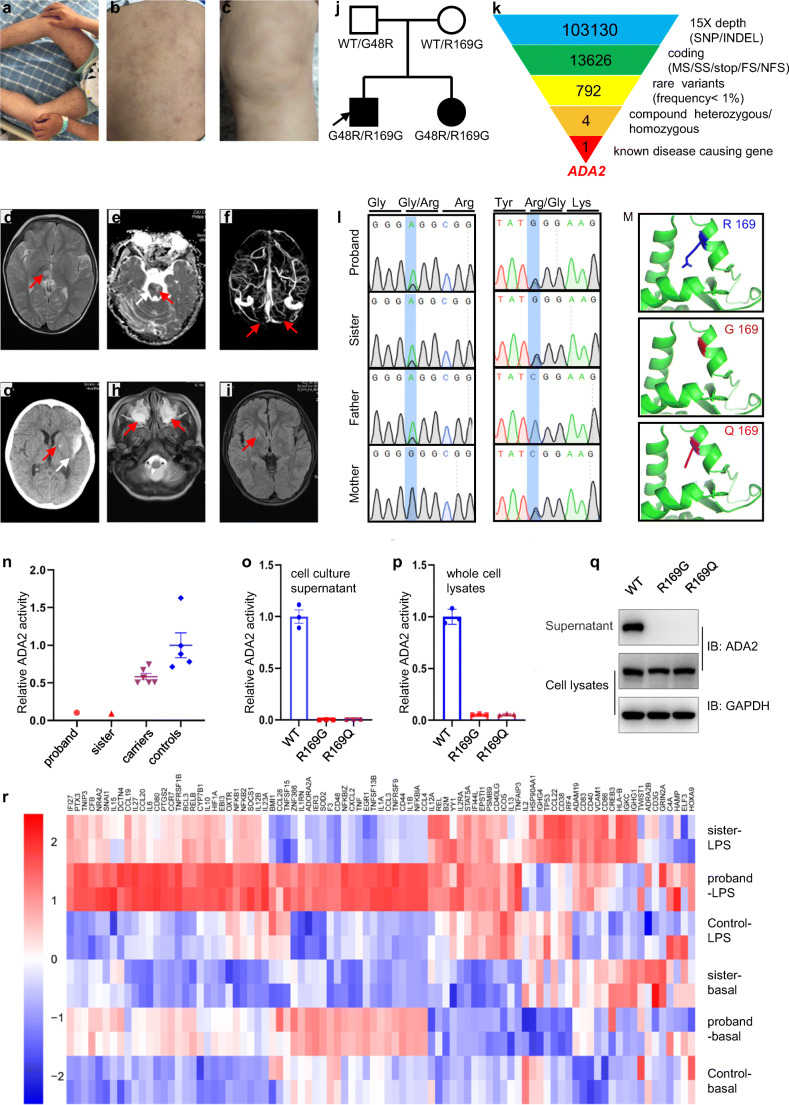
Clinical features of patients with DADA2 and identification of mutations in *ADA2*. **a–b** Livedo reticularis on the trunk and limbs of the proband. **c** Livedo reticularis on a leg of the sister. **d** Axial T2WI showed a high signal nodule in the right thalamus with clear edges, which was a fresh infarction area (red arrow). **e** ADC graph indicated a low signal patch area in the right part of the brainstem, which was a new infarct lesion (white arrow), and high signal patch shadow in the left part of the brainstem, which was an old infarction (red arrow). **f** 2d-MRV demonstrated bilateral transverse sinus narrowing, and local signal interruption, which suggested bilateral transverse sinus thrombosis (red arrow). **g** Calcification in the left globus pallidus (red arrow) and hemorrhagic stroke in the left temporal lobe (white arrow) were seen on CT image. **h** Axial T2WI showed Maxillary sinusitis (red arrow). **i** T2 dark-fluid indicated a new small lesion in the right lenticula (red arrow). **j** Pedigree of the family with compound heterozygous mutations in ADA2. **k** Schematic representation of the exome data-filtering approach assuming recessive inheritance in this family. **l** Confirmation of ADA2 mutations by Sanger sequencing. **m** In silico modeling of mutation at Arg169 based on crystal structure of human ADA2. **n** ADA2 enzyme activity of patients compared to carriers and healthy controls. **o–p** Relative ADA2 enzyme activity of wild type and mutant ADA2 in cell culture supernatant and whole cell lysates. **q** Western blot of wild type and indicated mutant ADA2 expression in supernatant and cell lysates. **r** RNA-sequencing analysis of genes involved in inflammatory pathway in patient PBMCs compared with age-matched healthy control (control-LPS, sister-LPS and proband-LPS: healthy control, sister and proband gene expression level with LPS stimulation(1 μg/ml); control-basal, sister-basal, proband-basal: healthy control, sister and proband gene expression at basal level)

Here we report the case of a 9yo Chinese boy who presented with a history of livedo reticularis (Fig. 1a, b) and recurrent fevers that began around 1 month of age. He had an ischemic stroke at 2yo and another at 8yo followed by a hemorrhagic stroke that same year. At the time of each of his strokes, he was treated with high-dose intravenous methylprednisolone, oral prednisone, and high-dose intravenous immunoglobulin (IVIG). However, he was still left gait instability, dysarthria, poor fine motor, and minor cognitive impairment. Laboratory work up showed elevated white blood cell counts and mild anemia as well as slightly elevated C-reactive protein (CRP) and erythrocyte sedimentation rate (ESR). Serum cytokines levels (including interleukin (IL)-2, IL-4, IL-6, IL-10, TNF-α, and interferon (IFN)-γ) were assessed using cytometric bead array techniques and IL-6 and IL-10 were found to be elevated (Table 1). T/B/NK cell counts by flow cytometry indicated that while the proportion of CD3^−^CD16^+^CD56^+^ NK cells was relatively low, the proportion of CD3^+^CD4^+^ T cells was relatively high and the ratio of CD3^+^CD4^+^ T cells and CD3^+^CD8^+^ T cells was also elevated. Serum immunoglobulin (Ig) and complement (C) testing revealed that IgM was notably low, while C4 was elevated (Table 1).

**Table 1:** Laboratory features of patients with DADA2

Laboratory features	Normal range	Brother						Sister
		Before Anti-TNF treatment					After Anti-TNF treatment	
		1M	2Y1M	7Y11M	8Y4M	8Y7M	10Y	4Y5M
CBC								
WBC (× 10^9^/L)	4.0–12.0	15.84↑	11.90	9.35	9.63	7.44	6.40	7.95
Hb (g/L)	110–155	91↓	105↓	109↓	92↓	96↓	126	103
PLT (× 10^9^/L)	100–400	535↑	453↑	327	328	239	209	334↓
CRP (mg/L)	0–8	23↑	12↑	3.52	8.85↑	32.45↑	0.53	14.08↑
ESR (mm/h)	0–20	47↑	13	40↑		18	8	49↑
Cytokines								
IL-2 (pg/mL)	1.1–9.8	2.1		1.3	2.1	1.1	1.0	3.5
IL-4 (pg/mL)	0.1–3.0	1.5		1.0	1.9	1.8	4.0	1.9
IL-6 (pg/mL)	1.7–16.6	35.2↑		22.0↑	1389↑	32.8↑	4.6	26.3↑
IL-10 (pg/mL)	2.6–4.9	7.0↑		3.7	6.4↑	8.0↑	6.1	5.9↑
TNF-α (pg/mL)	0.1–5.2	2.0		1.0	1.0	2.1	83.8	1.3
IFN-γ (pg/mL)	1.6–17.3	14.7		17.5↑	16.9	80.9↑	16.4	23.6↑
Ig^*^								
IgG (g/L)		5.44 (3.22–7.18)	12.16 (3.82–10.58)	15.9 (6.36–13.24)		9.2 (6.36–13.24)	9.50 (6.36–13.24)	18.2↑ (5.00–10.60)
IgA (g/L)		0.18 (0.13–0.35)	0.52 (0.14–1.14)	1.97 (0.63–1.79)		1.5 (0.63–1.79)	0.96 (0.63–1.79)	2.36 (0.34–1.38)
IgM (g/L)		0.06↓ (0.23–0.91)	0.29↓ (0.40–1.28)	0.27↓ (0.29–1.21)		0.15↓ (0.29–1.21)	0.30 (0.29–1.21)	0.6 (0.44–1.44)
IgE (IU/L)	1–100	13.5	99.9	56		18.9	42.1	54.4
C								
C3 (g/L)	0.9–1.8	1.43	1.35	1.41		1.40	1.10	1.66
C4 (g/L)	0.1–0.4	0.47↑	0.51↑	0.57↑		0.57↑	0.47	0.56↑
T/B/NK cells^*^								
CD19^+^ B cell (%)				27.15 (15.0–20.0)		13.50 (15.0–20.0)	18.00 (15.0–20.0)	24.10 (19.0–29.0)
CD3+ T cell (%)		68.72 (53.0–74.0)		67.5 (65.0–72.0)		76.8 (65.0–72.0)	65.00 (65.0–72.0)	63.00 (62.0–70.0)
CD3 + CD4+ T cell (%)		46.15 (36.0–38.0)		37.65↑ (27.0–34.0)		42.00↑ (27.0–34.0)	31.70 (27.0–34.0)	39.90 (30.0–40.0)
CD3^+^CD8^+^ T cell (%)		18.00 (13.5–19.5)		24.80 (23.0–30.0)		29.10 (23.0–30.0)	24.10 (23.0–30.0)	19.40 (20.0–27.0)
CD4^+^/CD8^+^		2.56↑ (1.9–2.5)		1.52↑ (1.0–1.4)		1.44↑ (1.0–1.4)	1.32 (1.0–1.4)	2.06 (1.2–2.0)
CD3^−^CD16^+^CD56^+^ NK cell (%)				3.15↓ (11.0–24.0)		10.2↓ (11.0–24.0)	7.20↓ (11.0–24.0)	8.10 (7.0–16.0)

^*^Because T/B/NK cell counts and serum IgG, IgA and IgM levels have different reference ranges at different ages, the corresponding reference ranges are indicated in parentheses following the data

Magnetic resonance imaging (MRI) showed abnormal signals in the right thalamus, which were determined to be inflammatory lesions (Fig. 1d). In addition, the MRI showed abnormal signals of medulla oblongata and pontine, and small softening foci in pons and right thalamus (Fig. 1e). Magnetic resonance venography (MRV) demonstrated bilateral transverse sinus narrowing, and local signal interruption, which suggested bilateral transverse sinus thrombosis (Fig. 1f). Computerized tomography (CT) detected a hemorrhagic lesion in left temporal lamella and calcification in the left globus pallidus (Fig. 1g). All imaging studies detected significant sinusitis (Fig. 1h).

Whole exome sequencing (WES) revealed two missense mutations in *ADA2*: c.50C > G (p.R169G) which was inherited maternally and c.142G > A (p. G48R) which was inherited paternally (Fig. 1j, k, l). These variants were also detected in his 3-year-old sister who appeared to be unaffected at the time of testing (Fig. 1l); however, 1 year later, she developed livedo reticularis on her face and limbs (Fig. 1c). Laboratory work up also showed mild anemia, mildly elevated CRP and ESR, and as well as elevated IL-6, IL-10, IFN-γ, IgG, IgA, and C4 (Table 1). MRI imaging has remained normal.

While p.R169Q is a well-known pathogenic variant in ADA2, p.R169G has not previously been reported in the literature. Structure modeling by PyMOL showed that it significantly altered the putative receptor-binging domain in a similar manner to R169Q (Fig. 1m). It was similar predicted to be deleterious/damaging by five in silico models including SIFT, PolyPhen-2, LRT, FATHMM, and PROVEAN. The p.G48R variant had been already reported in a Chinese literature by Wang et al. (http://rs.yiigle.com/CN141217201907/1156843.htm) and had been predicted to be pathogenic by several in silico models including SIFT, PolyPhen-2, LRT and PROVEAN.

Plasma ADA2 enzyme activity was found to be significantly decreased in the proband and his sister when compared to healthy age-matched controls (Fig. 1n) [7, 9]. To further determine the pathogenicity of R169G variant and R169Q variant on his own, we overexpressed wild type and mutant plasmid in HEK293T cells, and measured ADA2 enzyme activity in the medium and cell lysate 48 h after transfection. Both variants were found to decrease ADA2 activity in both the cell culture supernatant (Fig. 1o) and whole cell lysates (Fig. 1p). Additionally, the R169G and R169Q variants were found to affect the secretion of ADA2 into the cell supernatant (Fig. 1q).

We also studied the transcriptome profile of the siblings’ peripheral blood mononuclear cells (PBMCs) at baseline and upon lipopolysaccharide (LPS) stimulation (1 μg/ml) by RNA-seq. The proband’s PBMCs expressed increased levels of cytokines, including IL-6, TNF, IL-1β, and IL-10 both at a basal level and upon LPS stimulation when compared to an age-matched control (Fig. 1r), while his sister’s PBMCs only expressed moderately elevated IL-6 and IL-10 levels upon LPS stimulation compared to the healthy control (Fig. 1r).

Upon receiving the diagnosis, the proband began anti-TNF therapy (etanercept) [8]. He did not have another stroke after treatment. His fever quickly subsided on the second day of treatment. He was no longer anemic, and his CRP and ESR, and IL-6 had normalized at 3 months of treatment. But he was detected to have a new small lesion (Fig. 1i) on the brain upon MRI; however, the significance of this finding is unclear. After a year of treatment, his IgM also returned to normal level. His sister is still being followed; however, the family declined initiation of anti-TNF therapy for her.

## Discussion

Here we report a case of two siblings with novel variants in ADA2. The elder brother had typical clinical manifestations of DADA2, presenting with livedo reticularis, recurrent fevers, two ischemic strokes, and one hemorrhagic stroke with symptoms first reported around 1 month old. The younger sister was asymptomatic until she appeared livedo reticularis at 4Y3M old. This may be attributable to a combination of known clinical heterogeneity, even within families, and age-related penetrance [10].

One variant (p.R169G, c.505C > G) we reported in these siblings is novel. Additionally, the p.R169G and p.R169Q affected the secretion of ADA2 in which may result in inflammation and tissue damage. However, the exact pathogenicity of these two variants remains unknown. Considering p.R169Q is one of most common pathogenic mutations in DADA2, this might help for further study of the pathogenicity of this mutation and the precise treatment for patients with this mutation. Although another variant (p. G48R, c.142G > A) is a novel mutation in gnomAD, it had been already reported as a pathogenic mutation. What’s more this mutation was also collected in infevers (https://infevers.umai-montpellier.fr/web/detail_mutation.php) in the 3rd International Conference on Deficiency of ADA2.

Proinflammatory IL-6 and anti-inflammatory IL-10 cytokines were highly expressed in both siblings. This supports the hypothesis that M1 and M2 imbalance drives inflammation in DADA2 [2]. It should be noted that IL-10 is a pleiotropic cytokine. It can play an important role in immunosuppression through inhibition of macrophage activation, but excessive secretion of IL-10 in response to tissue damage can also increase inflammation. Because of this it is unclear if IL-10’s role in DADA2 is protective or destructive. There is difference between the cytokine levels and cytokine expression profile in PBMCs detected by RNA-seq. This might result from that the sister had no symptoms at the time when RNA-seq was performed. Although the serum TNF-α level was normal, the proband’s PBMCs expressed increased TNF and he was successfully treated by TNF inhibition. However, his sister had no increased TNF both on PBMCs and in serum. This confirms that TNF plays an important role in the pathogenesis of DADA2 and the transcriptome profile by RNA-seq is a useful and sensitive technique to detect inflammatory cytokines. This reminds us that it is better to determine the cytokine levels by multiple techniques.

The diagnosis of DADA2 in seemingly unaffected individuals through molecular testing can often be challenging for families and clinicians to manage. Treatment with TNF inhibitors has been proven to be remarkably effective in DADA2 patients, particularly when it comes to the prevention of strokes [8]. Many experts feel that treatment should be initiated in all molecularly detected patients to prevent symptoms from developing, unless therapy is contradicted as may be the case of patients presenting with immunodeficiency or bone marrow failure as a primary feature [4]. This case highlights the strong need for swift TNF inhibition as the proband had maintained fevers and elevated CRP on oral prednisone, and even had a stroke when the prednisone was tapered. However, his symptoms normalized and he has not had any significant strokes in the year since beginning treatment with TNF inhibition. The family opted out of treatment for the younger sister, given her relatively mild symptoms. While initiation of TNF inhibitors could prevent her from developing more serious symptoms, such as strokes, initiating therapy can burdensome for families, especially when it is unclear if an individual will ever develop more serious symptoms. Future guidance should be developed for treatment of pre-symptomatic, or asymptomatic, patients.

## Conclusions

Here we reported a pair of siblings with DADA2 caused by novel compound heterozygous variants in *ADA2*. On a clinical level, this case highlights the intrafamilial clinical heterogeneity of DADA2 and demonstrates some of the complexities of detecting the conditions in seemingly unaffected individuals. Early detection of DADA2 is critical and on a molecular level, this case contributes one novel variant (p.R169G) which may be detected in additional patients in the future. On a physiologic level, cytokine profiling revealed highly expressed levels of IL-6, IL-10, IL-1β, TNF, and IFN-γ which may play a role in DADA2 pathogenesis.

## Data Availability

All data generated or analyzed during this study are included in this published article.
